# Personal

**Published:** 1911-12

**Authors:** 


### PERSONAL

Dr. A. C. Hill of the State Health Department spent several days in November, visiting the schools of the Tonawanda Reservation, near Akron.

Dr. A. T. Bacon of Canaseraga was badly bruised and cut by being thrown from a hand car which jumped the track. A septic condition has developed but we trust that it will not prove serious.

Mr. C. J. Elberfield, Treasurer of the Memorial Hospital, Niagara Falls, reports good results from the Colonial Fair held in November.

Dr. George F. Cott of Buffalo, attended the Congress of Surgeons in Philadelphia in November.

Dr. Henry R. Harrower of Chicago, Editor of *Physiologic Therapeutics* and of *Successful Medicine* called on us during November.

Dr. John Gromann of Utica has returned from a three months' European trip, having visited many hospitals in England, France, Germany and Italy and having attended the Esperanto congress at Berlin.

Dr. Charles Van Bergen of Buffalo, gave an illustrated talk on his travels through the Holy Land, at the Church of the Good Shepard, November 6.

Dr. Frederick Frisch of Buffalo returned from a trip to Atlantic City, November 7.

Dr. Bertha Stoneman of Wellington, S. Africa, visited in Buffalo during November.

Dr. William H. Mansperger of Buffalo, has returned from a trip to Philadelphia and New York, having attended the Surgical Congress.

Dr. Nathalie Kavaleff Mankell has opened an office in the Professional Building, Buffalo, specializing in medical gymnastics and other physical methods of treatment.

Dr. Henry C. Buswell of Buffalo, entertained at dinner, at Statler's Hotel, Tuesday, November 14, in honor of Dr. Joseph

L. Miller of Chicago. The table was arranged in the form of a section of the hypophysis, in reference to the subject of Dr. Miller's paper before the Academy of Medicine.

---

Dr. J. C. Thompson of Buffalo, addressed the Mothers' Club in November.

---

Drs. M. P. Messinger and N. O. Brooks of Oakfield, were the guests of Dr. Benjamin A. Gipple of Alden, late in October.

---

Dr. George R. Stearns of Buffalo was in New York for a week about November 1.

---

Dr. Roswell Park of Buffalo gave a stereopticon lecture on the Hospital Knights of St. John of Jerusalem, before the University Club of Buffalo, October 28.

---

Dr. Jacob Goldberg of Buffalo has been appointed to membership on the municipal board of school examiners by Mayor Fuhrmann.

---

Dr. James Mooney of Buffalo will give a series of song recitals mainly of Schubert's compositions, at his home, during the winter. This is a pleasant and unusual phase of laryngology.

---

Dr. William Soch of Dunkirk attended the meeting of state health officers in New York, late in October.

---

Dr. George J. Eccle of Buffalo, has resigned as physician in charge of the Tuberculosis Dispensary, on account of pressure of private practice. Dr. Clarence L. Hyde, who has been Tuberculosis Inspector in the Health Department will succeed him but will spend a year in travel with leave of absence. During this time, the position will be filled by Dr. Harry N. Feltes.

---

Dr. William F. Jacobs will spend several months in California. At latest notice, he was the guest of Dr. William A. Dean of Ontario, Cal.

---

Dr. Edward B. Schwartz of Buffalo has moved to 1618 Fillmore Ave.

---

Dr. Harry M. Weed is in possession of the case-records of the late Dr. Alvin A. Hubbell.

Dr. H. R. Harrower of Chicago and Dr. A. Crandall Way of Perry Center have recently visited the editor.

---

Mayor Fuhrmann has most wisely reappointed Dr. Francis E. Fronczak, Health Commissioner of Buffalo, for five years. This is an honor which has been well earned by sound judgment, fearlessness and hard work.

---

Dr. Montgomery E. Leary of Rochester, was operated on November 20, for general peritonitis following a perforated appendix. At this writing he is still in critical condition.

---

Dr. H. N. Imboden of Rochester, formerly of Clifton Springs Sanatorium has been appointed Roentgenologist at Iola Sanatorium, the Monroe County Tuberculosis Hospital. *X*-Ray examinations of the lungs will be a special feature of the clinical investigation of the cases.

---

Dr. John R. Williams gave an address, well illustrated with lantern slides, on Modern Conceptions of the Physiology of Digestion before the Rochester Academy of Science on November 27.

---

Dr. Edward Clark of Buffalo and Dr. B. W. Sherwood of Syracuse are the speakers at a joint meeting of all the physicians of Monroe County at East High School, Rochester, N. Y., on Saturday, December 2. The meeting is held at the suggestion of the State Health Department and is followed by a Public Mass Meeting in the evening.

---

Dr. Myron A. King of Rochester, is recovering from an operation for the relief of a strangulated hernia.

---

Dr. Helen P. Morehouse-Kennedy of Buffalo, is attending lectures in the University of Munich as a "hörer."

Buffalo is well represented at the University of Berlin. Drs. Carroll J. Roberts, Charles A. Bentz, John L. Eckel and Frank A. Valanti are attending and report that they are much pleased with the opportunities for work.

---

Dr. L. H. Smith of E. Aurora, spent part of November at Clifton Springs.

---

Dr. Charles E. Welch of Westfield, returned November 4, from a trip to Denver.

